# Prostate Artery Embolization in the Treatment of Massive Intractable Bleeding from Prostatic Neoplasms: A Case Report and Systematic Review

**DOI:** 10.3390/jcm13010065

**Published:** 2023-12-22

**Authors:** Lorenzo Moramarco, Antonino M. Grande, Maurizio Vertemati, Paolo Aseni

**Affiliations:** 1 Radiologia—Neuroradiologia Diagnostica ed Interventistica, Fondazione IRCCS Policlinico San Matteo, 27100 Pavia, Italy; dottorlo@yahoo.it; 2 Divisione Cardiochirurgia, Fondazione IRCCS Policlinico San Matteo, 27100 Pavia, Italy; amgrande@libero.it; 3 Department of Biomedical and Clinical Sciences “L. Sacco”, Università degli Studi di Milano, 20157 Milan, Italy; maurizio.vertemati@unimi.it; 4 Dipartimento di Emergenza Urgenza, ASST Grande Ospedale Metropolitano Niguarda Hospital, 20162 Milan, Italy

**Keywords:** prostatic neoplasms, prostatic carcinoma, benign prostatic hyperplasia, hematuria, angiography, prostate artery embolization, urinary bladder hemorrhage

## Abstract

Lower urinary tract symptoms (LUTS) and hematuria are common symptoms in men with neoplasms, mainly affecting the elderly population. Prostatic arterial embolization (PAE) is a minimally invasive procedure that has shown promising results in managing LUTS and massive intractable prostatic hematuria in patients with benign prostatic hyperplasia (BPH) and prostate cancer (PCa). A few studies, however, have provided valuable insights into the durability and efficacy of PAE focusing on the long-term effectiveness, quality of life, and cancer-specific control of hemostasis and urinary symptoms. As a result of concomitant cardiovascular conditions, these patients often take anticoagulants or antithrombotics, which can worsen their hematuria and clinical status. Transurethral resection of the prostate (TURP) is considered a very high-risk procedure, even without massive bleeding, and requires discontinuation of vitamin K antagonists and antiplatelet therapies. Such patients usually have their surgery postponed, and PAE should be considered a safe alternative treatment. We aimed to report a narrative review from 1976 to June 2023 of the current state of PAE for massive and intractable hematuria, highlighting recent developments in this technique, including prospective cohort studies, and focusing on long-term outcome, safety, and complication management of patients with prostatic neoplasms who develop significant hemorrhagic symptoms. Additionally, we present a case report and a simple algorithm for treating intractable bleeding in a 92-year-old man with PCa and massive hematuria.

## 1. Introduction

After lung cancer, prostate carcinoma (PCa) is a worldwide health problem, being the second most frequent malignancy in men, with 1,276,106 newly diagnosed cases and causing 358,989 deaths (3.8% of all deaths caused by cancer in men) in 2018 [1]. Death rates for PCa have been decreasing, and this is mainly due to earlier diagnosis because of screening and improved treatment, resulting in a real postponement of death for some men with metastatic disease and often in a consequent variation in the attribution of cause of death [2]. Metastatic prostate carcinoma presents significant morbidity and rapidly worsens the quality of life. More than 30% of these patients will necessitate lower urinary tract surgery, and in the late stages of the disease, more than 25% will require indwelling bladder catheterization (IBC) insertion through the urethra or suprapubic, or palliative transurethral resection of the prostate (TURP) [3,4]. PCa and benign prostatic hyperplasia (BPH) represent two distinct yet interconnected urological conditions that significantly impact the male population. These disorders are notorious for their potential to induce acute lower urinary tract symptoms (LUTS) that can have profound implications for patients’ health and quality of life. Among these symptoms, urinary retention requiring IBC and urinary tract hemorrhage stand out as particularly alarming clinical manifestations, often necessitating immediate medical intervention and even emergency care.

While these conditions differ in their underlying pathology and clinical course, they share a common thread in the potential to precipitate acute LUTS that can be severe and life-threatening, especially in the elderly population with multimorbidity.

TURP is considered the surgical gold standard for patients with severe symptomatic BPH. It is effective and durable, but it can cause several complications, including bleeding, dilutional hyponatremia, sexual dysfunction, and incontinence [5]. Moreover, TURP is a “high risk of bleeding” procedure that, in high-risk patients, involves discontinuation of anticoagulant/antiplatelet therapy while PAE is considered a safe treatment alternative for high-risk patients on anticoagulants. These features boosted the development of less invasive treatment options for BPH: holmium laser enucleation of the prostate, thulium laser-based enucleation, GreenLight laser therapy, and transurethral water vaporizing therapy. However, none of these treatment options has shown superior clinical benefit when compared with TURP [6].

In 1976, Mitchell reported selective hypogastric embolization in four patients experiencing severe prostatic hemorrhage, after undergoing biopsy or prostatectomy, with high success rates [7]. The first successful therapeutic PAE for bleeding in a PCa patient was reported in 1977 by Bischoff and Goertler [8] using Gelfoam; later, Nadalini [9] described 14 cases of hypogastric arteries embolized with isobutyl-2-cyanoacrylate for hemorrhage due to bladder and prostatic carcinomas. Prostatic artery embolization (PAE) was later used in high-surgical-risk patients with acute urinary retention who had IBC, and eventually emerged as a potential innovative technique for selected patients as a minimally invasive alternative treatment to TURP in patients with moderate to severe BPH determining urinary tract symptoms and/or bladder outlet obstruction. In all patients with LUTS, PAE proved to be a minimally invasive treatment option for BPH and was shown to be safe and effective in reducing prostatic volume and improving LUTS relating to BPH [10,11] with a low morbidity rate and lower incidence of sexual dysfunction due to retrograde ejaculation [12].

In 1990, Li [13] performed PAE in BPH in a group of 16 patients with an age range of 44–72 years. Nine patients had bladder cancer, one had prostatic cancer, and six had BPH. A combination of Gelfoam (Pharmacia & Upjohn, Kalamazoo, MI, USA) and coils were employed to embolize the internal iliac arteries, and hemostasis was achieved in 15 patients without complications. Since then, transarterial PAE of symptomatic BPH has slowly gained popularity. This is because it is minimally invasive, does not require general anesthesia, and appears to be effective in stopping bleeding and relieving voiding difficulties. In 2000, DeMeritt [14] reported a case of PAE performed with polyvinyl alcohol particles for hematuria associated with BPH: the patient had symptomatic improvement, hematuria was immediately stopped, and the prostate size was reduced by 52% of the initial size in the first 5-month follow-up and 62% of the initial size at the 12-month follow-up. In 2010, Carnevale [15] reported PAE using microspheres in two patients as a primary treatment for acute urinary retention due to BPH. For elderly men with symptomatic BPH, PAE can be an alternative treatment: it is performed by a femoral artery puncture and under conscious sedation instead of general anesthesia. PAE produces an immediate infarction of the central gland, resulting in its overall volume decrease; delayed fibrosis produces a further size reduction. Moreover, in BPH, the transitional zone, the noncancerous growth of the prostate surrounding the prostatic urethra, becomes ischemic. This process is also extended in the peripheral zone, suggesting a potential clinical role as a palliative treatment for prostate carcinoma [16]. However, despite the available data, PAE has yet to be established as a standard-of-care treatment option for patients with LUTS and low urinary tract bleeding due to BPH/PCa.

In this study, we aimed to perform a systematic literature review on indications and outcomes of PAE in the treatment of refractory bleeding from the lower urinary tract, especially focusing on the management of refractory bleeding from prostatic adenocarcinoma. In addition, we present a simple treatment algorithm based on a challenging case report of a patient with multimorbidity who presented with massive refractory bleeding from prostatic adenocarcinoma and developed massive refractory hematuria, which was successfully treated with PAE.

## 2. PAE and the Importance of Careful Evaluation of the Prostate Gland’s Vascular Supply

**Figure 1:**
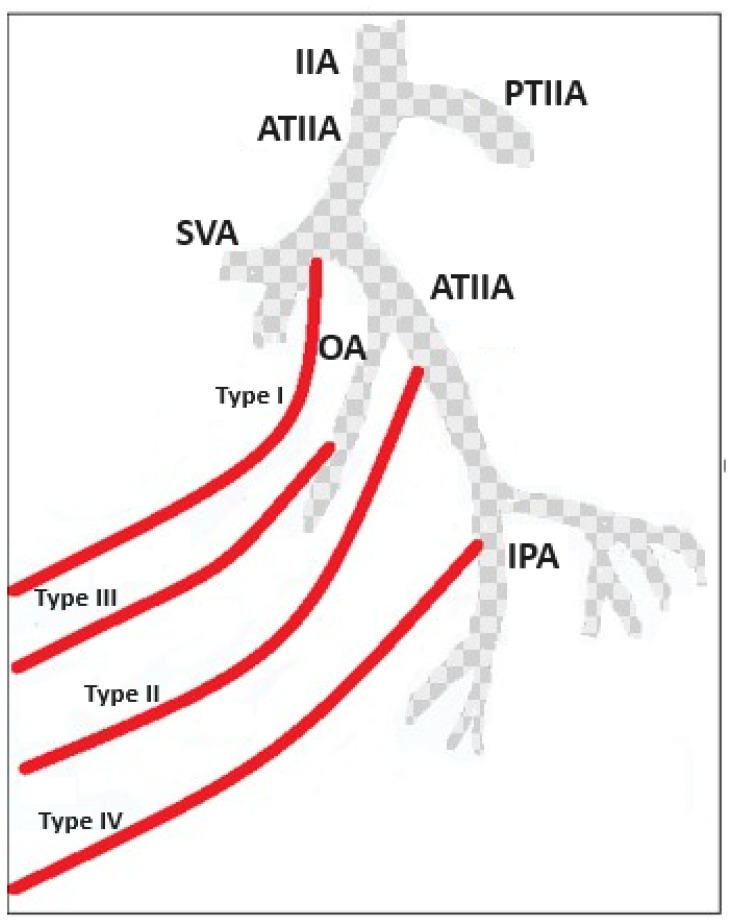
Prostate arteries (PAs) have common origins and anastomoses with feeding arteries of critical pelvic structures: understanding prostatic arterial supply is necessary to predict non-target embolization risk. IIA = internal iliac artery, ATIIA = anterior trunk internal iliac artery, PTIIA = posterior trunk internal iliac artery, SVA = superior vesical artery, OA = obturator artery, IPA = internal pudendal artery; in red are the type I–IV PA origin.

When planning PAE, it is vital to carefully consider the anatomical connections of the prostate gland [17]. There are two prostatic arteries (left and right), also known as the inferior vesical arteries, and other arteries that supply structures and organs in the male pelvic region. As a result, unintended embolization may occur in non-target areas. The inferior vesical artery (IVA), which supplies blood to the bladder, is situated in the pelvic area. It branches off from the internal iliac artery, usually alongside the middle rectal artery within the anterior division. Its blood flow reaches the bladder fundus. The inferior vesical artery supplies blood to the prostate and seminal vesicles in males, like the vaginal artery in females. In addition, this artery can share a trunk with the superior gluteal and internal pudendal arteries, and it can also branch off from the internal pudendal artery. In most cases, there is only one additional branch, but this varies from individual to individual. The IVA also supplies the ductus deferens, a segment of the spermatic passageway. Regarding the prostatic arteries, their origins can significantly differ between the left and right sides of the body and among patients. Most commonly, they originate from the internal pudendal artery. The prostate has a dual arterial supply: the cranial or vesico-prostatic artery (sometimes called the anterior-lateral prostatic pedicle) and the caudal prostatic artery (known as the posterior-lateral prostatic pedicle). In cases with only one prostatic artery (occurring in 60% of cases), both prostatic pedicles may arise from the same artery. However, in patients with two independent prostatic arteries (occurring in 40% of cases), the pedicles originate separately. On the other hand, the posterior-lateral prostatic pedicle has an inferior or distal origin, providing blood to the peripheral and caudal glands. It may be closely associated with rectal or anal branches. Careful evaluation of the prostate’s vascular anatomy is crucial for the successful planning of PAE. It is significant to note that up to 60% of cases exhibit significant connections between the prostatic branches and surrounding arteries. These connections should be carefully considered when planning an embolization. Figure 1 shows a schematic illustration of the main anatomical variations in bladder and prostatic vascularization.

The prostatic artery (Pa) presents highly variable origins. De Assis et al. has suggested five types of anatomic origins of PAs, which have been classified as follows [17]:

Type I: PA originating from the anterior division of the IIA, in a common trunk with the SVA 28.7%,

Type II: PA originating from the anterior division of the IIA, inferior to the SVA 14.7%,

Type III: PA originating from the obturator artery 18.9%,

Type IV: PA originating from the IPA 31.1%,

Type V (others): less common origins 5.6%. This subgroup includes the “corona mortis” (crown of death), a connection between the obturator and the external iliac artery or vein. It is located behind the superior pubic ramus at a variable distance from the symphysis pubis (range 40–96 mm). The name “corona mortis” suggests that a significant hemorrhage may occur if accidentally cut and it is difficult to achieve subsequent hemostasis [18].

## 3. Materials and Methods

### Research Methodology for Literature Review

**Figure 2:**
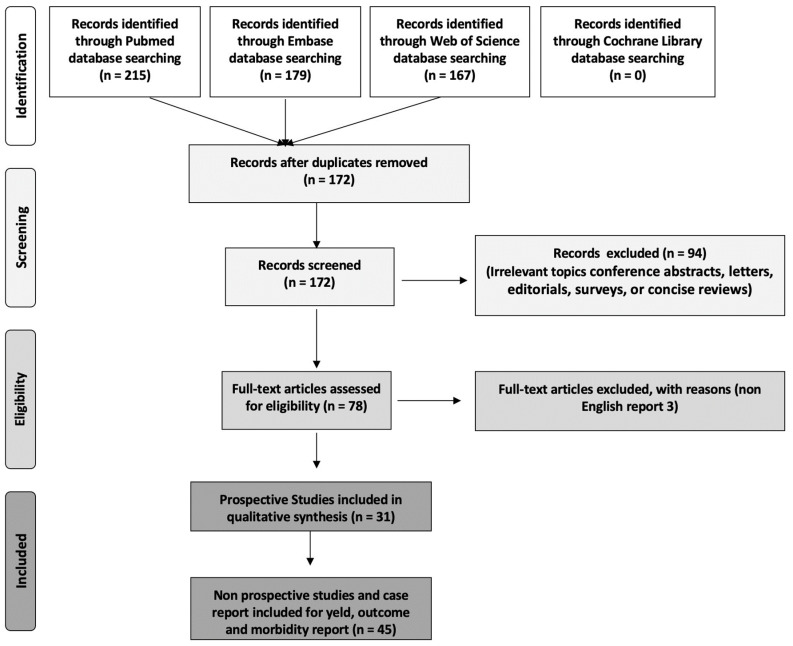
PRISMA flow chart diagram.

This literature review adheres to the Preferred Reporting Items for Systematic Reviews and Meta-Analysis (PRISMA) guidelines, although due to the nature of this review, not all items on the checklist were applicable. The search was conducted across multiple databases, including PubMed, EMBASE, Web of Science, and Cochrane, from 1976 to June 2023. Our search employed MeSH terms, including “prostatic artery embolization” AND “hematuria”. Following the removal of duplicate entries, we meticulously screened the remaining articles based on their titles and abstracts. For inclusion in this review, we considered all peer-reviewed articles published in the English language that involved patients with hematuria stemming from PCa or BPH and who had undergone angioembolization to control bleeding (see Figure 2 PRISMA flowchart). As a subsequent step, we searched for publications containing prospective clinical trials of PAE for the management of massive intractable bleeding originating from PCa. All prospective clinical trials were extracted and classified according to qualitative synthesis in a dedicated table.

This study falls outside the scope of typical interventions covered by registration databases, and our systematic review was not previously registered due to the nature of the research and the specific characteristics of the study population based on a very narrow and unique patient group, such as individuals with massive intractable bleeding from prostatic adenocarcinoma who have undergone prostatic artery embolization (PAE). Our study is more of an in-depth analysis rather than a confirmatory investigation, and therefore, pre-registration might not provide substantial benefits.

## 4. Results

As a result of our search, we were able to find 211 articles in EMBASE, 80 in PubMed, and 167 in Web of Science; no article was found in the Cochrane database. A total of 73 papers were excluded as conference abstracts, letters, editorials, surveys, or concise reviews. After eliminating duplicate articles, we meticulously analysed 96 relevant publications (see Figure 2). From this pool, we identified and synthesized data from 31 prospective studies (8 of 31 comparative) exclusively focused on prostatic artery embolization. In light of the high prevalence of BPH in the general population, it has been found that there are fewer prospective studies that have been conducted on patients who have been treated with PAE for Pca.

In addition, we synthesized data from the remaining 66 non-prospective studies and case reports. All 31 prospective studies are comprehensively presented and summarized in Table 1 for detailed examination.

Table 1 presents the first author, year of research, region, age, number of patients treated, angioembolization material, outcomes for patients, and some critical points highlighted by the authors.

A summary of the remaining 66 articles about PAE for bleeding and LUTS secondary to PCa was provided and reported on the following topics: efficacy, yield, morbidity and complications, patient selection, long-term outcomes, and comparison with other treatments.

### 4.1. Efficacy of PAE

PAE has shown promising efficacy in controlling bleeding and improving LUTS in patients with PCa. Saro et al. [47] confirm the effectiveness and safety of PAE in elderly patients aged ≥ 80 years old (mean 85.29, range 80–98). There were significant improvements in the International Prostate Symptom Score (IPSS) and quality of life (QoL), showing that PAE is a feasible low-risk treatment for prostatic hematuria also in elderly patients with or without urinary retention. Several prospective studies were carried out to evaluate PAE for acute urinary retention and/or prostates larger than 80 mL, which are not typically eligible for TURP. In these studies, the procedure proved to be safe and effective also in larger prostates, with reported clinical success in 72.4–98% of patients [9,17,21,29,30]. PAE’s efficacy in achieving hemostasis has also been demonstrated specifically in patients with massive hematuria associated with PCa. Embolization of the prostatic arteries reduces bleeding episodes by targeting the vascular supply of the neoplastic lesions. The success of PAE in treating massive hematuria can be attributed to its ability to selectively target the tumor’s prostatic vasculature.

### 4.2. Yield of PAE

The yield of PAE in terms of bleeding control generally appears favorable and varies among studies, from 67% [23], where patients had PCa and refractory hematuria, to 100% [41]. It is critical to recognize a success rate of 94.1% in patients aged > 80 years old [31]. Success rates may depend on the patient population, the severity of bleeding, and the expertise of the interventional radiologists performing the procedure. Several studies emphasize the importance of careful patient selection for PAE in the context of prostatic neoplasms and massive hematuria by carefully evaluating the underlying vascular anatomy, tailoring the procedure to individual patient characteristics, anatomical variations, and anastomotic shunting to reduce complications.

### 4.3. Morbidity and Complications

Anyway, PAE is generally considered a minimally invasive procedure with a low rate of major complications. Informed consent should include a further discussion of the rare but potentially serious complications of nontarget embolization to the penis, rectum, and bladder [49]. Minor complications include post-PAE syndrome, dysuria, hematuria, and hematospermia. In our review, four cases with major complications of partial bladder necrosis, one sepsis, and two cases of penile ulcers, all requiring surgery, have been reported [9,33,34,40]. Access-site complications are described and include puncture size hematoma (PSH) and femoral artery dissection (FAD); four (1.85%) FAD cases and four (1.85%) PSH cases were reported by Ray [34], one (1.3%) FAD case by Insausti [48], and six PSH cases (11.8%) by Brown [39]. Postembolization syndrome (i.e., gluteal pain, fever, nausea, emesis) has been reported as manageable with a symptomatic treatment approach only, with complete resolution within a few days. As compared with superselective embolization as distal as possible, embolization of the main trunk of the internal iliac artery or the whole anterior or posterior division of the iliac artery increased the risk of ischemic complications.

### 4.4. Patient Selection for PAE

Proper patient selection is crucial to the success of PAE. Patients should be carefully evaluated to determine if they are suitable candidates for the procedure. PAE in a non-emergency setting is typically considered for patients who have failed conservative management and are not candidates for more invasive treatments. Moreover, it is necessary to have a detailed understanding of the anatomy of the pelvic arteries to safely perform the procedure, avoiding nontarget embolization [49]. In the emergency setting in patients with recurrent or intractable bleeding secondary to Pca hemorrhage, PAE should be considered the preferred therapeutic indication, and also in those patients who will be candidates for a definitive surgical treatment; detection of active bleeding by CT scan or angiography is not an obligatory finding to proceed with PAE in patients with refractory cancer hemorrhage. A standardized approach to imaging protocols for pre-procedural assessment to accurately identify the prostatic neoplasm, assess its vascularity, and plan the embolization procedure should be discussed. A multidisciplinary approach involving urologists, interventional radiologists, oncologists, and other relevant specialists for a comprehensive evaluation of each patient can be crucial in tailoring the most appropriate treatment option based on the individual patient’s characteristics. To ensure consistency across embolization procedures, advanced imaging techniques, such as dynamic contrast-enhanced MRI or 3D angiography to improve pre-procedural planning, are crucial. Identifying potential complications and implementing additional interventions should be part of regular follow-up assessments.

### 4.5. Long-Term Outcomes

Long-term outcome data in PCa patients show that PAE successfully treats associated complications such as LUTS, urinary retention, and hematuria with a low risk of serious adverse events [49]. PAE as the primary oncological treatment for PCa is currently inadequate [42]: more large-scale randomized trials are needed for further assessment of PAE as a potential option for a combination treatment for prostate cancer.

### 4.6. Comparison to Other Treatments

PAE can be an alternative to other treatments, especially in BPH LUTS refractory to medical therapy. It is necessary to underline that α-blockers and 5α-reductase inhibitors are associated with adverse side effects associated with a decrease in sexual function and QoL [50]. We found several studies [9,10,29,48] where a comparison PAE/medical therapy in BPH has been performed, especially in patients with LUTS refractory to pharmacological treatment, where QoL was significantly improved. Furthermore, PAE has been compared to other treatments such as surgical interventions, radiation therapy, or cystoprostatectomy in patients with massive intractable bleeding from PCa. Several studies have compared PAE and TURP in patients with LUTS, showing promising results in patients treated with PAE, including rates of resolution of urinary tract symptoms, with significant reductions in the International Prostate Symptom Score (IPSS), improvement in peak urinary flow (Q_max_), and overall lower complication rates [34,42,48]. PAE and TURP are minimally invasive therapeutic options. It is well known that TURP is highly effective in relieving symptoms associated with prostatic neoplasms and improving quality of life, and this observation is supported by several clinical trials (Table 1). It has been shown that TURP is highly effective at reducing prostate volume, improving flow rates, and resolving hematuria. Because TURP can provide rapid relief from obstructive symptoms, it should be the preferred therapeutic option for those patients fit for surgery with severe LUTS. However, TURP is also associated with some specific complications, such as retrograde ejaculation, bleeding, and catheterization after surgery. TURP’s invasiveness can result in a higher perioperative complication rate and longer recovery times than PAE. The less invasive nature of PAE renders the procedure the preferred option for those patients with associated poor clinical conditions or in anticoagulation therapy. The shorter recovery time associated with PAE makes it an attractive option for patients seeking quicker recovery. The limited long-term data on PAE compared with TURP require further prospective studies to establish symptom relief durability. When deciding between TURP and PAE, it is crucial to emphasize the importance of a patient-centered approach that takes individual preferences, comorbidities, and treatment goals into consideration.

## 5. A Suggested Algorithm and a Demonstrative Case Report

**Figure 3:**
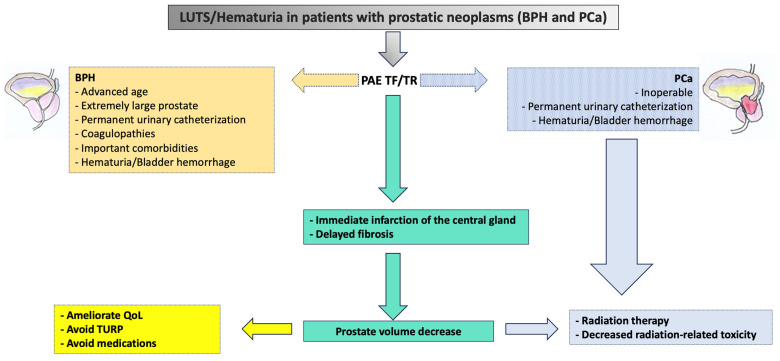
A simple algorithm to evaluate prostatic artery embolization in patients with LUTS and prostate cancer when surgery is not an option. TF = transfemoral, TR = transradial, LUTS = low urinary tract symptoms, BPH = benign prostate hyperplasia, PCa = prostate carcinoma, QoL = quality of life, TURP = transurethral resection of the prostate.

In Figure 3, we propose a simple algorithm that can be used to quickly submit patients for PAE when surgery is not an option. It can also provide a brief overview of what can be done after the embolization procedure. This causes an immediate infarction of the central gland, a decrease in prostate volume, and resolution of symptoms. The patients are then followed up in two different clinical settings, whether they have BPH or PCa.

**Figure 4:**
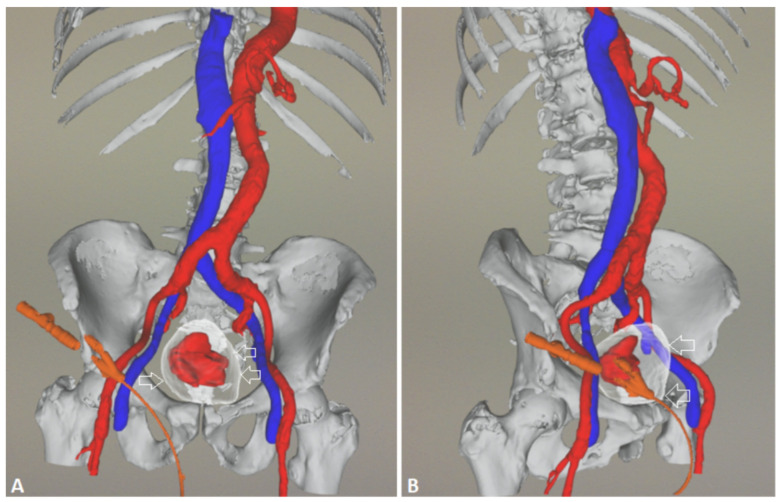
Angio-CT 3D, (**A**) frontal view, (**B**) right oblique anterior view; empty arrows indicate the bladder, with a huge hypervascular tumoral mass inside stained in red. The patient-specific 3D models were displayed using ARTICOR^®^ (Artiness srl, Milano, Italy) and obtained using a marching cube algorithm.

**Figure 5:**
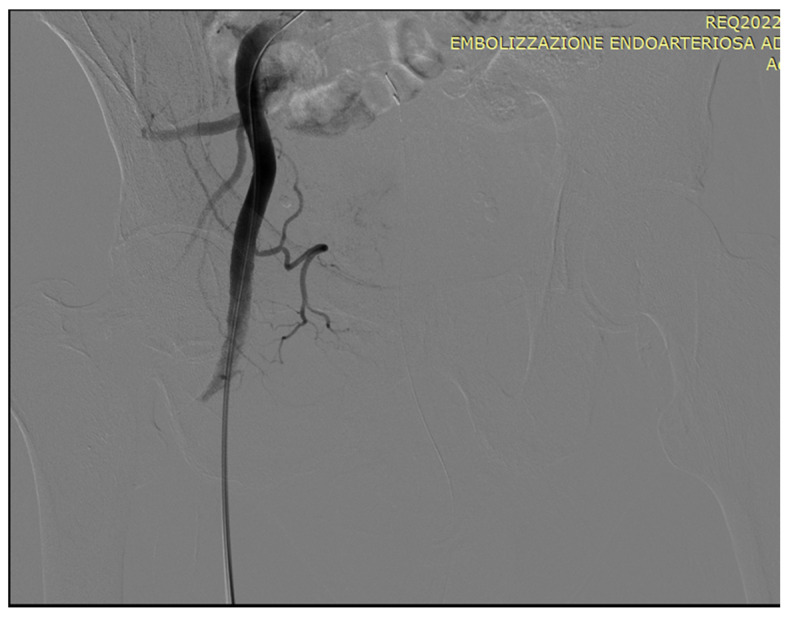
Pelvic angiography to evaluate the iliac vessels: a 5F sheath is placed in the right common femoral artery under local anesthesia.

**Figure 6:**
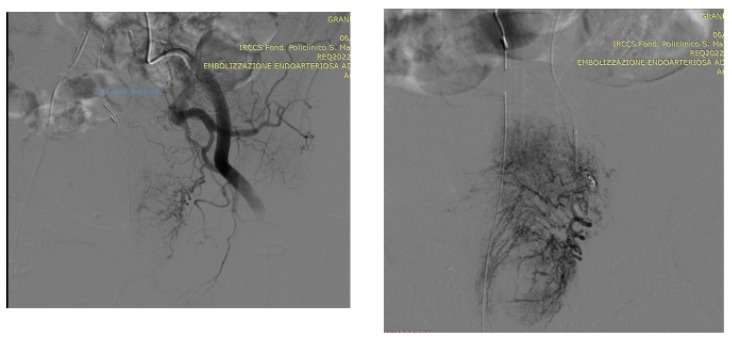
A 5F pigtail catheter is advanced into the abdominal aorta to the level of the iliac bifurcation, and then the left prostatic artery is selected; DSA (digital subtraction angiography) image following superselective microcatheterization of the left prostatic artery, which appears hypertrophied and is seen arising from the anterior division of the internal iliac artery via a common vesicoprostatic trunk; note extravasation of contrast medium from branches of the left prostatic artery before treatment.

**Figure 7:**
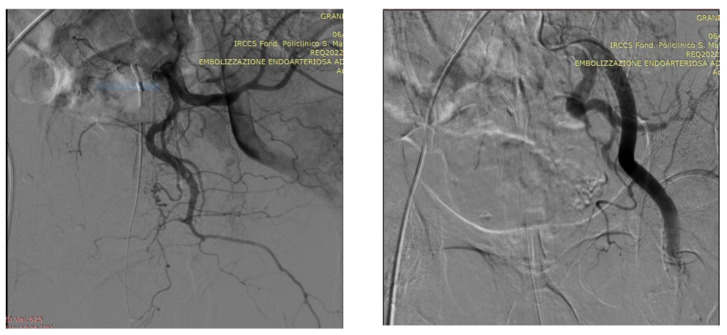
Left prostatic artery embolization (PAE) performed using the standard proximal embolization first, followed by the distal (PErFecTED) technique [26]; PAE was obtained by injecting a solution of microbeads (Embosphere 300–500 μm, Merit Medical, South Jordan, UT, USA) mixed with 9 mL of saline and 9 mL of contrast medium.

**Figure 8:**
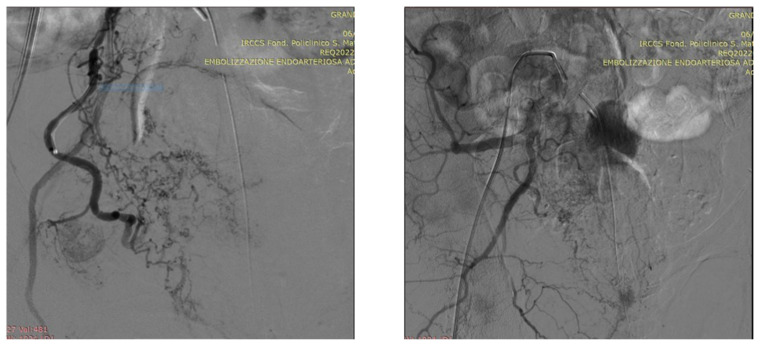
DSA (digital subtraction angiography) image following superselective microcatheterization of the right prostatic artery, appearing extremely hypertrophied.

**Figure 9:**
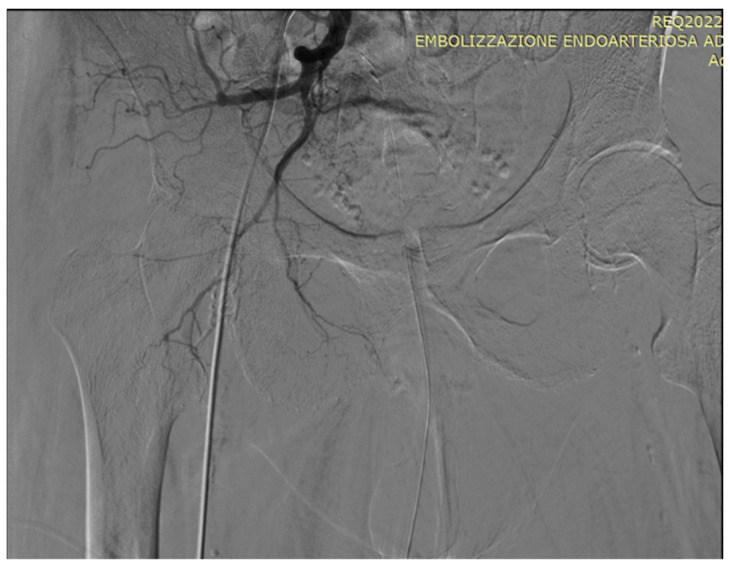
Right PAE result.

As an illustration of our proposed algorithm (Figure 3), we describe a clinical complex case report in a patient with LUFT who underwent PAE as a rescue procedure for intractable urinary bleeding from his prostate cancer. A 92-year-old man, affected by PCa and with a long-term indwelling bladder catheter (IBC), was admitted for a massive bladder hemorrhage. The patient, a former smoker, had blood hypertension treated with an ACE inhibitor; he presented with elevated PSA levels of 62 ng/mL, and his symptomatology was limited to nocturia causing 2–3 nocturnal lifts. A prostatic biopsy revealed a Gleason score of 7 (3 + 4) and adenocarcinoma in 6 of 12 specimens. Laboratory blood test values were within the reference range; chest X-ray, bone scan, and thoracic and abdominal CT scan were negative. The chest CT scan showed initial pulmonary fibrosis as an incidental finding. Because of the advanced stage of the disease and pulmonary fibrosis, the primary treatment modality was hormonal therapy: so, he assumed Bicalutamide 50 mg/day and Triptorelin, a synthetic agonist analog of gonadotropin-releasing hormone (GnRH), 11.25 mg/every 3 months. After 6 months, Triptorelin was discontinued, and only Bicalutamide 150 mg/day was prescribed. In March 2019, he underwent Choline PET-CT, demonstrating common iliac and aortic nodal chains with metastatic involvement, and in June, his PSA was 20.78 ng/mL. In January 2020, his PSA was 27 ng/mL; it increased to 38 ng/mL in May, and in August the value was 53 ng/mL; testosterone was <0.1 ng/mL. At this point, a Choline PET scan showed several skeletal secondary localizations in the dorsal and lumbar vertebral bodies, the pelvis, and the 11th right rib. In September 2020, Enzalutamide 160 mg daily was started, and he continued Triptorelin 3.75 mg monthly: one month later PSA was 20.47 ng/mL and in January 2021 the value was 19.24 ng/mL. Although PSA decreased, the patient had urinary retention, and an IBC was positioned and replaced every four weeks. In May 2021, PSA reached 44 ng/mL, Enzalutamide was stopped and Abiraterone, 1000 mg daily, and Prednisone 5 mg × 2 daily, were started continuing Triptorelin. In July 2021, PSA was 72 ng/mL and in November the value climbed to 334 ng/mL: the oncologist replaced Abiraterone with Megestrol, 160 mg daily, continuing Triptorelin. In January 2022, the patient started to have hematuria that rapidly became a gross hemorrhage with clot formation and urinary obstruction. In addition to urinary obstruction and massive hemorrhage, the patient also suffered three syncopal episodes. He was immediately hospitalized, and a three-way IBC was positioned for continuous bladder irrigation to prevent blood clots. His Hb dropped from 14.5 g/dL to 7.9 g/dL, and a blood transfusion was done; despite continuous bladder irrigation, blood was still present in the urine. Chest and abdominal CT scans revealed large bleeding associated with PCa expansion into the bladder, involving the neck and trigone (Figure 4). To stop the bleeding, a PAE was performed. The procedure is described in Figure 5, Figure 6, Figure 7, Figure 8 and Figure 9. Hematuria was resolved one week after the procedure, LUTS ameliorated, and quality of life improved significantly. The patient died at the age of 93 in the fourth post-procedure month of respiratory insufficiency due to pulmonary fibrosis.

### Technical Details of PAE

In an angio-suite, a 5 Fr valved sheath was inserted into the right common femoral artery. Under direct fluoroscopic guidance, selective catheterization of the internal iliac arteries was carried out with a 5 Fr pre-curved catheter vertebral. PAE was performed using the standard “Proximal Embolization First, Then, Embolize Distal” (PErFecTED) technique [26].

Prostatic arteries showed bilateral origin type 2. After a superselective catheterization of both prostatic arteries with a microcatheter 2.0 F (Progreat, Terumo), PAE was completed using the PErFecTED technique by injecting a solution of microbeads (Embosphere 300–500 μm, Merit Medical) mixed with 9 mL of saline and 9 mL of contrast medium. The procedure was safe and effective, resulting in the complete embolization of the prostatic arteries. Finally, groin hemostasis was achieved with Angioseal 6 Fr. Hematuria was resolved one week after the procedure, LUTS ameliorated, and quality of life improved significantly.

## 6. Discussion

The management of patients with high-risk, early-stage PCa represents a major challenge for all disciplines involved in the treatment of this common malignant neoplasm.

Pisco and Coll [40] made preliminary evidence of the technical feasibility and safety of prostatic artery chemoembolization (PACE) for treating PCa: in their prospective study, 20 PCa patients underwent PACE; the mean Gleason score range was 6 to 10, and their staging was T2N0M0. PACE was performed with a combination of Chelidonium majus extract, docetaxel, and 150–300-µm Embosphere particles. All patients were treated on an outpatient basis and discharged home the same day. Technical success of the procedure, defined as bilateral PAE, was achieved in 16 out of 20 patients. Adverse events were few and mostly minor. A multiparametric prostate MRI done at 12 months for the 10 patients with biochemical successes showed that of the 7 patients with a Gleason score of 6, no changes were seen in the lesions, whereas the 3 patients with a Gleason score of >7 had >50% tumor size reduction. Peacock’s prospective study demonstrated that PAE is a clinically significant adjunctive therapy for alleviating LUTS and achieving significant volume reduction before RT, resulting in decreased radiation-related toxicity from prostate alone RT for PCa [44]. According to the Society of Interventional Radiology, PAE might lead to minor and major complications. Side effects of embolization, i.e., pain, hematuria, hematospermia, urethral burning, rectal bleeding, urinary tract infection, balanitis, hematoma, diarrhea, dissection acute urinary retention, non-target embolization. Non-target embolization complications have been categorized as major complications depending on the necessity of the therapy, overnight admission, or prolonged hospitalization.

The present systematic review collected the available data attributable to prostate arterial embolization in patients with prostate cancer. We analysed and assessed comparative and non-comparative publications. Not all focused only on the patients with prostate cancer. Nevertheless, the heterogeneity of the population showed the wide range of indications and effectiveness of this method. The studies prove the procedure is safe, burdened with a low risk of complications, and accomplishes technical and clinical success. In selecting the optimal treatment method in patients unfit for surgery, the minimally invasive method has to be considered. Thereby, prostate artery embolization for patients with PCa experiencing massive hematuria is a promising option with an important impact on the quality of life as pain reduction, improvement in urinary symptoms, and overall well-being and is now becoming a part of the standard-of-care treatment algorithm for patients with urinary hemorrhage and other sequelae secondary to prostate cancer.

In this article, we also tried to provide a systematic review of PAE, delving into some intricate aspects and clinical challenges in those patients with prostatic cancer associated with important comorbidities.

Based on a detailed analysis of the 31 prospective studies reported in Table 1, several factors contribute to the heterogeneity of cohorts of patients. First, demographic variability is evident. Although age is not a significant variable, comorbidities, severity of prostatic neoplasm, and different embolization techniques used across the different studies examined are evident (see Table 1). In addition, the criteria used to include and exclude patients from the studies varied greatly. All these factors can significantly impact the generalizability of results. Variations in pre-procedural patient preparation, post-procedure care, and follow-up protocols are lacking in the majority of studies examined. Other quantitative outcome metrics, such as changes in prostate size, are infrequently reported in the prospective studies. Improvement in LUTS and the reduction in bleeding episodes are regularly reported and seem comparable. However, with the limitations of unknown time-dependence, only eight studies compared PAE with TURP to confirm its efficacy. Studies differ greatly in the duration of follow-up in all prospective studies, which can impact conclusions on long-term results. Studies report adverse events for PAE with some variation in severity, but with homogenous conclusions about their low incidence.

A simple algorithm was developed from our experience that can be utilized in the emergency setting as a life-saving procedure in case of massive hematuria, showing the essential strategy for timely and effective management of prostatic symptoms. Simple algorithms can be useful in emergency clinical practice with a possible positive impact on patient outcomes. PAE can be performed on an outpatient basis and usually does not require IBC unless the patient has urinary retention [51,52] and can be considered a good alternative to the standard TURP [53,54].

Concomitant ischemic heart disease and low left ventricle ejection fraction are not a contraindication to PAE, and several case reports have been reported in these high-risk patients with important LUTS improvement [55,56,57]. In patients with severe pain and bleeding caused by advanced prostate cancer, PAE appears as one of the most reliable and advantageous options [58].

PAE is a minimally invasive technique associated with a high success rate of hemostasis and a low incidence of recurrence [59]. In patients with patent carpal circulation, PAE can be successfully performed via transradial access [60,61]. In the context of prostate cancer and massive hematuria, a collaborative approach may contribute to better outcomes and care for these patients. For this reason, multidisciplinary collaboration in the management of prostatic cancer patients with massive hematuria involving interventional radiologists, urologists, and oncologists is of the utmost importance. Limitations of this review may be attributed to the inclusion criteria and search strings selected for this review, which limit the inclusion of potentially relevant research. Other papers such as case reports and studies in non-English literature could have raised other relevant issues. Furthermore, using other databases might have included additional studies and reflected the current literature more broadly. Despite adherence to PRISMA guidelines, variations in data extraction may introduce errors and biases in the synthesis processes. Inclusion criteria with different designs (e.g., observational studies, monocentric clinical trials, multicentric clinical trials) may introduce multiple biases and are more prone to confounding factors. Some studies may not have reported all relevant outcomes, leading to incomplete data synthesis, and potentially impacting the review’s precision and accuracy. High heterogeneity among the included studies in our review in terms of populations, interventions, and outcomes hampers the possibility to perform a structured meta-analysis, which can usually allow reliable statistical conclusions.

## 7. Conclusions

PAE is a minimally invasive approach consisting of the occlusion of the prostatic arteries performed under fluoroscopic guidance by trained interventional radiologists. Although there are too few comparative/prospective studies, PAE presents a particularly relevant role in patients with prostatic carcinoma or BPH in patients with LUTS and massive hematuria. Unlike more invasive surgical interventions, PAE has shown promise in managing LUTS and massive hematuria in elderly and fragile patients, especially those taking anticoagulants or antithrombotics. Almost all studies examined emphasize the importance of careful patient selection for PAE in the context of prostatic neoplasms and massive hematuria by careful evaluation of the underlying vascular anatomy, tailoring the procedure to individual patient characteristics enhancing the likelihood of success and minimizing the risk of complications.

## Figures and Tables

**Table 1:** Comparative, prospective studies in prostatic artery embolization (PAE) reporting: the name of the first author, the year of the study, the region where the study was performed, the age and number of patients treated, the material used for angioembolization, outcomes of patients, and relevant points highlighted by the authors. Legend: IPSS = International Prostate Symptom Score, FU = follow-up, TS = technical success, CF = clinical failure, CS = clinical success, COMP/PROSP = comparative/prospective study, OP = open prostatectomy, BIL = bilateral, UNI = unilateral, ATH = atherosclerosis, HEM = hematuria, UI = urinary infection, UR = urinary retention, AUR = acute urinary retention, M = months, UB = urethra burning, PV = prostate volume, PVR = post-void residual, IIEF = international index erectile function, QoL = quality of life, ED = erectile dysfunction, FAD = femoral artery dissection, PES = post-embolization syndrome, HS = hospital stay, IBC = indwelling bladder catheter, RHOPA = refractory hematuria of prostatic origin, MRI = magnetic resonance imaging, PSH = puncture size hematoma, DYS = dysuria, HEMS = hemospermia, PACE = prostatic artery chemoembolization, HT = hormonal therapy, SDYS = sexual dysfunction, BF = biochemical failure, BS = biochemical success, NK = no known, RT = radiotherapy. – = data unavailable. The arrows means decrease and increase.

Author Year	Country	Type Study	N.°	Age, Mean	Particles μm	Results	Compl./Notes
Pisco 2013 [11]	Portugal	PROSP in BPH LUTS refractory to medical therapy	89	74.1	180–300	TS 97% 86/89. FU 12 M. At 1 M IPSS, QoL, PVR, IIEF improved, all *p* < 0.01.	1 bladder wall necrosis that needed surgery
Bilhmt 2013 [19]	Portugal	PROSP in BPH to verify particle size effects 100 μm (A) vs. 200 μm (B)	80	63.9	100–200	FU 6 M. No significant differences were found in pain scores. A had a greater ↓ in PV (8.75 cm^3^ *p* < 0.13), PSA level (2.09 ng/mL *p* < 0.001); B had greater ↓ in IPSS (3.64 points *p* < 0.052) and QoL (0.57 points *p* < 0.07).	No significant differences were found in adverse events between 2 groups.
Gao 2014 [20]	China	COMP/PROSP in BPH LUTS PAE/TURP	PAE57 TURP57	67.7, 66.4	355–500	TS TURP 100% PAE 94.7%, CF 3.9%/9.4%. FU 24 M. IPSS, QoL, Qmax, PVR, PV, PSA had significant improvements in both groups.	TURP had greater improvements in IPSS, QoL, Qmax, and PVR at 1 and 3 M, and greater ↓ in PSA and PV when compared with the PAE group (*p* < 0.05).
Kurbatov 2014 [21]	Italy Russia	PROSP in BPH	88	66.4	NK	FU 12 M	IPSS QoL Qmax PVR PV *p* < 0.05
Bagla 2014 [22]	U.S.A.	PROSP in BPH	20	66.6	100–200	TS 90%, 10% due to ATH, Significant ↓ IPSS QoL	No minor/major compl.
Russo 2015 [23]	Italy Russia	COMP/PROSP PAE/OP	80	67	300–500	FU 12 M OP had lower IPSS *p* < 0.05, PVR lower *p* < 0.05, higher PF *p* < 0.01; PAE showed higher Hb, shorter HS and IBC time.	PAE could be a feasible minimally invasive technique but failed to demonstrate superiority to OP because of the increased risk of persistent symptoms and low PF after 1 year.
de Assis 2015 [17]	Brazil	PROSP in BPH LUTS in PV > 90 g	35	64.8	NK	FU 3 M: mean PV ↓ from 135.1 g to 91.9 g *p* < 0.0001, IPSS and QoL improved *p* < 0.001.	A significant negative correlation was observed between PSA at 24 h after PAE and IPSS 3 months after PAE (*p* = 0.0057): excessively elevated PSA within 24 h is associated with lower IPSS.
Li 2015 [24]	China		24	74.5	50–100	TS 92% Bil 86%, UNI 14% due to ATH IPSS QoL PVR *p* < 0.002 PV *p* < 0.001	No major complications. AUR 32% HEM 14% UB 36%
Carnevale 2016 [25]	Brazil	COMP/PROSP in BPH TURP/PAE/Perf	PAE15 Perf15 TURP15	63.5 60.4 66.4	NK	IPSS, QoL, PV and Qmax significantly improved. TURP and Perf both had significantly lower IPSS than PAE but not significantly different from one another. TURP had significantly higher Qmax and smaller PV but required spinal anesthesia and ↑ HS.	Perf = Perfected Proximal Embolization First, Then Embolize Distal [26]
Wang 2016 [27]	China	COMP/PROSP 2 groups for mean PV: A 129/B 64 mL	115	71.5	100	TS A 93.8% B 96.8%. FU 12 M. Better outcome in larger PV	IPSS QoL Qmax PVRV IIEF PSA PV significantly improved in both groups.
Gabr 2016 [28]	Saudi Arabia	PROSP in BPH LUTS UR and IBC	22	72.5	300–500	TS 100%	FU 9 M: IPSS Qmax PV PSA *p* < 0.001 No major compl.
Pisco 2016 [29]	Portugal	PROSP in BPH LUTS refractory to therapy	630	65.1	NK	TS 98.1% BIL 92.6% UNI 7.4% Clinical success rates at 1–3 y and 3–6.5 y were 81.9% and 76.3%	IPSS QoL Qmax PV PSA IIEF PVR *p* < 0.001
Isaacson 2016 [30]	U.S.A.	PROSP BPH LUTS	12	69	NK	TS 100%. FU 3 M: mean improvements in IPSS and QoL were 18.3 points (5–27) and 3.6 points (1–6), respectively.	7 cases transfemoral access, 5 cases transradial access. No major compl., no ischemic injuries.
Yu 2017 [31]	China	COMP/PROSP in BPH PAE in AUR and IBC weaning vs. relieving LUTS without AUR	27	66	100–300	PAE BIL 100% IBC removed in 14/16 87.5%	Outcome comparable to cases without AUR No periprocedural compl.
Chen 2017 [32]	Korea Taiwan	PROSP in PCa stage 4 Refractory HEM	9	71.9	NK	FU 3 M: 2 recurrent HEM, 4 died no PAE related, 3 no HEM	No complications
Mordasini 2018 [33]	Switzerland	Prosp to provide PAE tumoricidal effect in PCa patients	12	45–75	100	Complete necrosis in 2, partial in 5, viable cancer cells in all 12	Partial bladder wall necrosis in 2 requiring surgery
Ray 2018 [34]	UK	COMP/PROSP in BPH PAE/TURP	PAE216 TURP89	66	NK	PAE is clinically effective, producing a median 10-point IPSS improvement from baseline at 12 M while TURP has a median 15-point improvement. TURP HS is significantly longer than PAE.	PAE compl.: sepsis 1, blood transfusion 1, FAD 4, PSH 4, penile ulcers 2. PAE provides significant improvement in IPSS and QoL, although some of these improvements are greater in the TURP arm.
Abt [35] 2018	Switzerland	COMP/PROSP BPH PAE 48/TURP 51	99	PAE 65.7 TURP 66.1	250–400	FU 3 M: PAE and TURP show similar results	PAE BIL 75% UNI 25% PAE HS 2.2/TURP HS 4.2 *p* < 0.001
Maclean 2018 [36]	UK	PROSP in BPH to study clinical outcome PAE and PV	86	64.9	NK	UNI/BIL TS% 100/96.5	No major compl. Initial PV and %PV reduction at 3 M predict good clinical outcomes at 12 M.
Salem 2018 [37]	U.S.A.	PROSP in BPH LUTS	45	67	NK	FU at 1-3-6-12 M IPSS QoL Qmax *p* < 0.001 PVR at 6 M *p* 0.02, at 12 M *p* 0.025; PV ↓ *p* 0.001	Minor compl.: dysuria 13, HEM 6, HEMS 2, urinary frequency 3 and UR 2.
Franiel 2018 [38]	Germany	PROSP in BPH to study MRI predictors of clinical success	30	66	250	TS 90% 27/30 BIL in 24 (89%). Significant MRI predictors of clinical success were not identified.	FU 1-3-6 M: IPSS < 18 with ↓ > 25%, QoL score < 4 with ↓ ≥ 1, Qmax ≥ 15 mL/s and ↑ ≥ 3.0 mL/s) rates: 59% (16/27), 63% (17/27), 74% (20/27).
Brown 2018 [39]	Australia	PROSP in BPH LUTS (40), HEM (1), IBC (10)	51	67.8	250	BIL 92.2% UNI 7.8% FU 3 M: IPSS, QoL, Qmax, PV *p* < 0.001; PVR *p* < 0.018. 7 cases 70% had IBC removal.	PSH 11.8%, DYS 84.3%, perineal pain 25.5%, HEMS 11.8%, fever 9.8%, 1 medial uni gluteal irritation, 1 transient rectal hemorrhagic spot.
Pisco 2018 [40]	Portugal	PROSP PACE in PCa staging T2N0M0, 15 refused surgery, 5 wanted to stop HT	20	67.5	150–300	TS 80%, 16/20. BF 18.7%, 3/16 (PSA ↓ to < 2 ng/mL followed by PSA ↑ to >2 ng/mL within 1 month after success). BS at 12–18 M was 62.5%, 10/16.	FU 12–18 M: 1 small bladder wall necrosis removed by surgery, 2 UR, 2 SDYS, all recovered. PACE allowed a biochemical response and is a promising treatment.
Thulasidasan 2019 [41]	UK	PROSP to study PAE BPH LUTS or RHOPA	159	70	100–200	TS 98% IBC removal in 13/24 in retention. PAE controlled HEM in 12/12 RHOPA cases.	The highest baseline IPSS and reduction in PV on the 1st MRI present the most benefit from PEA.
Mailling 2019 [42]	Denmark	Prosp in advanced PCa LUTS 9, UR 6 cases	11	75.8	300–500	TS 93.3%, 1 case unsuccessful due to ATH, bilateral 10/15; IPSS reduced 12.2 points	4 cases did not have Fu: 2 died, 1 lost, 1 not done for Ather.
Rampoldi 2019 [43]	Italy	PROSP in BPH, IBC in all cases	43	77.9	300–500	BIL 76.7%, UNI 18%, 4.7% not done for ATH. IBC removal in 80.5%	TPV reduced *p* < 0.001 UI 3/7.5%, UR 6/14.6%
Peacock 2020 [44]	U.S.A.	PROSP PEA before RT in PCa LUTS	9	71	300–500	FU 18 M in 5 that had RT at the same center. Mean IPSS after PEA 13.8 *p* < 0.02, mean PV ↓ was 23.1%. No BF.	PAE is a clinically significant adjunctive therapy for alleviating LUTS and achieving significant volume reduction before RT, resulting in decreased radiation-related toxicity from prostate alone RT for PCa.
Insausti 2020 [45]	Spain	COMP/PROSP in BPH PAE/TURP	PAE 23 TURP 22	-	300–500	FU 12 M: PAE had IPSS ↓ *p* < 0.08 and better QoL *p* < 0.002; PV ↓ was better in TURP *p* < 0.001.	PAE group had fewer compl. 15/47 (TURP) *p* < 0.001.
Tapping 2021 [46]	UK	PROSP in BPH symptoms refractory to medical therapy	50	67	200–500	TS 96% 48/50. FU 24 M. IPSS at 24 M ↓ *p* < 0.001, PV ↓ at 3 and 12 M but not significantly different at 24 M.	Initial PV was not a good predictor of CS.
Saro 2022 [47]	UK	PROSP in BPH and PCa LUTS and HEM	54	85.29	180–300	TS 92.6%. 30 surgery was contraindicated, no possible for ATH 4. IPSS and QoL significantly improved at 12 and 24 M. PAE was successful in 19 out of 20 with IBC for UR.	17 patients, 4 PCa, had HEM: PAE resulted in CS in 16 with immediate bleeding stoppage. PV ↓ significantly within 6 M. IBC removal successful in 16 out of 17. No intra-or postprocedural compl. were encountered.
Insausti 2022	[48] Spain	PROSP in BPH LUTS refractory to therapy	81	73.87	400 ± 75	TS 100% BIL 85.2% UNI 14.8%: 3 cases impossibility PA cannulation, 4 PA perfused rectum or penis, 5 ATH. CS 78.5%	FU 12 M: IPSS Q = L Qmax *p* < 0.01, PVR < 0.05 Compl. 11 cases: 3 UI 3 UR, 3 PES, 1 ED, 1 FAD.

## Data Availability

All data are available following the search methodology described in methods section.

## Abbreviations

PCa = prostate carcinoma, IBC = indwelling bladder catheterization, BPH = benign prostatic hyperplasia, PAE = prostatic artery embolization, TURP = transurethral resection of the prostate, LUTS = acute lower urinary tract symptoms, PA = prostatic artery, PAs = prostatic arteries, IIA = internal iliac artery, ATIIA = anterior trunk internal iliac artery, PTIIA = posterior trunk internal iliac artery, SVA = superior vesical artery, OA = obturator artery, IPA = internal pudendal artery, FU = follow-up, TS = technical success, CF = clinical failure, CS = clinical success, COMP/PROSP = comparative/prospective study, OP = open prostatectomy, BIL = bilateral, UNI = unilateral, ATH = atherosclerosis, HEM = hematuria, UI = urinary infection, UR = urinary retention, AUR = acute urinary retention, M = months, UB = urethra burning, PV = prostate volume, PVR = post-void residual, IIEF = international index erectile function, IPSS = International Prostate Symptom Score, QoL = quality of life, ED = erectile dysfunction, FAD = femoral artery dissection, PES = post-embolization syndrome, HS = hospital stay, IBC = indwelling bladder catheter, RHOPA = refractory hematuria of prostatic origin, MRI = magnetic resonance imaging, PSH = puncture size hematoma, DYS = dysuria, HEMS = hemospermia, PACE = prostatic artery chemoembolization, HT = hormonal therapy, SDYS = sexual dysfunction, BF = biochemical failure, BS = biochemical success, NK = no known, RT = radiotherapy.
